# *Tph2^−/−^* female mice restore socio-sexual recognition through upregulating ERα and OTR genes in the amygdala

**DOI:** 10.1371/journal.pone.0193395

**Published:** 2018-02-22

**Authors:** Ying Huo, Yaohua Zhang, Huifen Guo, Yingjuan Liu, Qi Fang, Jianxu Zhang

**Affiliations:** State Key Laboratory of Integrated Management of Pest Insects and Rodents in Agriculture, Institute of Zoology, Chinese Academy of Sciences, Beijing, China; Nanjing University, CHINA

## Abstract

The central 5-hydroxytryptamine system impairs sociosexual behaviors and olfaction preferences in sexually naive mice. However, it remains unknown whether reproductive experiences impart an effect on the sexual olfactory preferences of female mice lacking central serotonin. Here, we aimed at examining such effects and the underlying mechanisms using *Tph2* knockout female mice. Sexually naive *Tph2*^*−/−*^ female mice failed to recognize olfactory cues regarding sex, genetic relatedness, and social hierarchy despite exhibiting normal olfactory discrimination. However, reproduction-experienced *Tph2*^*−/−*^ female mice recovered sexual olfactory preferences, as did sexually naive *Tph2*^*+/+*^ females. Meanwhile, both the estrogen receptor α and oxytocin receptor in the amygdala of reproduction-experienced *Tph2*^*−/−*^ females presented upregulated expression at the mRNA level and an upward tendency at the protein level vs. sexually naive *Tph2*^*−/−*^ females. Intracerebroventricular administration of a combination of estrogen receptor α and oxytocin receptor agonists, but not either agent alone, could restore the sexual olfactory preferences of sexually naive *Tph2*^*−/−*^ female mice to some degree. We speculate that estrogen receptor α and oxytocin receptor activation in the amygdala after reproductive experiences restores sexual olfactory recognition in *Tph2*^*−/−*^ female mice.

## Introduction

In rodents, choice of mate relies heavily on olfactory signals, of which urine odor is one of the most important signals with which to communicate sex, social status, kinship, and individual identity [1, 2, 3]. For high reproductive success and offspring fitness, females usually use olfactory assessment to ensure accurate sex identification and to select genetically dissimilar and high-quality male mates [4, 5, 6, 7].

Sexual experiences are able to guide animals toward stimuli that are predictive of sexual reward and reproductive success [8, 9, 10, 11]. In particular, sexual experiences can provide a disinhibitory influence on the disruptive effects of certain treatments, such as anosmia, castration, penile deafferentation, novelty stress, and deficiency in olfactory information processing [8, 12, 13]. Under natural conditions, sexual behaviors usually result in the occurrence of reproduction, including copulation, pregnancy, parturition, and lactation in females [10, 11]. Therefore, reproductive experiences might also play a disinhibitory role in sexual behaviors.

As one of the most important neurotransmitters in the brain, serotonin (5-hydroxytryptamine; 5-HT) has a widespread and profound effect on information processing, cognition, memory, stress adaption, anxiety, depression, aggression, sexual preference, and maternal care in rodents [14, 15, 16, 17, 18, 19]. Tryptophan hydroxylase 2 (*Tph2*) is the initial and rate-limiting enzyme on the pathway from tryptophan to central 5-HT [14, 16]. *Tph2* knockout mice generated by deleting exon 5 that encodes the tryptophan hydroxylase domain only have minute amounts of central 5-HT and show strong deficiencies, such as growth retardation, 50% lethality in the first 4 weeks after birth, loss of sexual preference evidenced by the paradigms of mating choice assay, genital odor preference assay, bedding preference assay, and lordosis assay, exaggerated aggression, and decreased anxiety [14, 15, 17, 18, 19].

Evidence for both the disinhibitory effect of reproductive experiences and the inhibitory effect of central 5-HT deficiency on sexual preferences of both male and female mice has been published [10, 11, 12, 13, 14, 15]. So, we speculated that reproductive experiences could mitigate the detrimental effects of *Tph2* knockout on the sexual olfactory preference. Furthermore, females are often the choosy sex that could discriminate amongst competitive males inmate choice for high fitness [15, 20]. Then, reproductive experience would make the *Tph2* knockout female mice prefer female urine odor more than the male one? The right strain? The dominant male odor?

Even though there are still no direct evidences of how ERα, ERβ, OT and OTR changed in *Tph2* knockout mice, a sophisticated interplay among 5-HT, estrogens and oxytocin (OT) has been shown to affect sexual performance [21, 22, 23, 24]. Estrogen acts largely through estrogen receptor α (ERα) and estrogen receptor β (ERβ) to alter the function of the 5-HT system at various levels, including synthesis, reuptake, neural firing, degradation, binding, and receptor activation [22, 23]. Furthermore, estrogen modulates OT and oxytocin receptor (OTR) via ERα and ERβ, and may also contribute to regulate sexual preferences [22, 24, 25, 26]. ERβ controls OT production in the paraventricular nucleus (PVN) of the hypothalamus. Then, axonal projections of the PVN neurons reach the amygdala, where ERα drives the transcription of the OTR gene and ultimately regulates sexual preferences [24, 27].

Here, we examined whether reproductive experiences could make the *Tph2* knockout female mice prefer female urine odor more than the male one? The right strain? The dominant male odor? And, the role of ERα, ERβ, OT and OTR played in such processes?

## Materials and methods

### Experimental animals

The *Tph2* line generated from C57BL/6 mice was a generous gift from the laboratory of Dr. Yi Rao (Peking University, Beijing, China). It was then maintained and genotyped in our laboratory, as previously described [6]. About 150 female and 80 male heterozygotes were repeatedly used for breeding and about 2,000 pups were got. These pups were weaned at the age of 3 weeks, and approximately 1-cm sections of the tail of each mouse were collected at the same time for isolation of genomic DNA. The dams and pups were monitored about every 2 weeks. After the pups reached the age of 8 weeks, the male mice were housed singly, while female mice sharing the same genotype were kept in groups of four or five until treatment. The pups (about 0.5%) showing obvious signs of illness were euthanized.

Twenty male and 24 female C57BL/6 mice at an age of 40 weeks were used as urine donors for sex. The estrous status of the female C57BL/6 mice was determined by microscopic examination of vaginal epithelium. An additional 20 male C57BL/6 mice and 20 male Balb/c mice at an age of 12 weeks were used as urine donors for genetic relatedness. Nine pairs of male C57BL/6 mice with a stable dominance–submission relationship were selected as urine donors for social hierarchy at the age of 17 weeks. The formation of a stable dominance–submission relationship was ensured by use of a dyadic encountering test in a neutral area between paired males over the course of 21 consecutive days at the age of 13 weeks. In brief, weight-matched male mice were paired and simultaneously placed into a clean mouse cage for 10 min of continuous recording beginning with initial aggressive behavior (i.e., tail rattles, sideway postures, pushing, chasing, and biting) [5, 28].

The plastic cage was 27 × 12 × 17 cm in size. The housing room was kept under a reversed 14L: 10D light/dark photoperiod (lights on at 7:00 pm), and the temperature was maintained at 23 ± 2 °C. Commercial standard rat/mouse pellet chow (Beijing KeAo Feed Co., Beijing, China) and water were provided *ad libitum*.

### Ethical notes

All of the mice experiments were reviewed and approved by the Ethics Committee of the Institute of Zoology, Chinese Academy of Sciences (approval No. IOZ12018). Adequate measures were taken to minimize discomfort for mice and to ensure that the entire experimental protocol complied with the Institutional Guidelines for Animal Use and Care at the Institute of Zoology, Chinese Academy of Sciences.

### Reproductive experiences

Eight sexually naive *Tph2*^*+/+*^ female mice (aged 19–20 weeks) and 27 *Tph2*^*−/−*^ female mice (aged 12 weeks) were separately brought into the home cage of *Tph2*^*+/+*^ male mice of the same age for sexual intercourse. When there was a visible sign of pregnancy, the male mice were removed from the cage. The gestation period showed no difference between *Tph2*^*+/+*^ and *Tph2*^*−/−*^ females. Then, the female mice were allowed to experience parturition and lactation. In spite of being fertile and producing milk, *Tph2*^*−/−*^ female mice present poor maternal care leading to a low survival rate of their pups, most of which die on Day 2 or 3 after birth. If the pups died during lactation, the corpses were immediately removed from the cage. If the pups were still alive, they were removed from the cage on the day following 21 days of lactation. Whether or not there were living pups, the female mice had to go all the way through to 21 days of lactation. Female mice experienced the processes including mating, pregnancy, parturition, and lactation were classified as reproduction-experienced (R-E) females. R-E female mice were housed singly for 1–2 weeks before the start of behavioral tests.

### Urine collection

As previously described [3], during the dark phase, we placed each donor in a clean mouse cage (27 × 12 × 17 cm) covered with a wire grid 1 cm above the bottom of the cage. The urine was immediately drawn after the animal urinated, and transferred to an Eppendorf tube in ice using a disposable glass capillary (i.d. 1.8 mm; length, 15 cm). If the urine was deposited next to feces, it was not collected due to possible contamination. The urine was stored at –20 °C until use.

### Binary test of urinary attractiveness

Binary choice tests using the capillary method were carried out to explore urinary preference [5, 6]. The recipient subjects were investigated regarding their preference for two tested urine samples during the dark phase in their home cages. Urine samples were presented to the test recipient subjects using the same two disposable glass capillaries (internal diameter, 1.8 mm; length, 15 cm), both containing 2 μL urine approximately 1 cm from the sample-containing end, with the other end sealed by odorless gum suspending the sample aliquot inside the capillary. After showing an initial sniffing response, the mouse investigating behavior was recorded for 3 min. The length of time that the female mice spent sniffing within 1 cm from the tip and licking the end of the capillary was recorded using two handheld stopwatches. Every new test was performed with new capillary tubes and urine samples. The estrous cycles were not determined in any test for recipient female mice.

**Fig 1:**
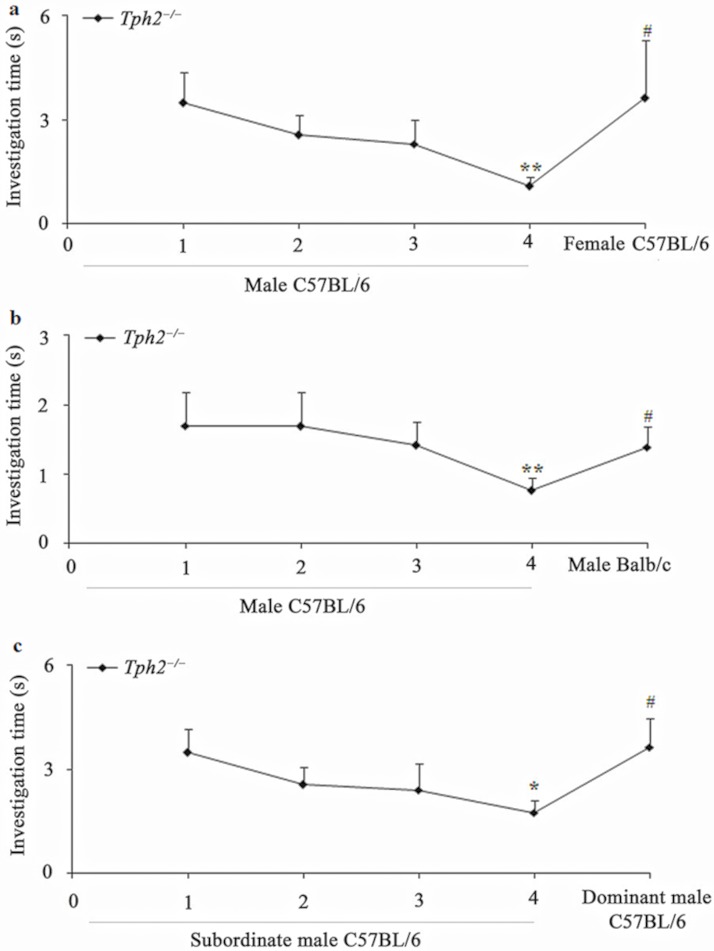
Discrimination (mean ± SE) of 12 sexually naive *Tph2*^*−/−*^ female mice between urine odor related to sex, genetic relatedness, and social hierarchy. (a) Male C57BL/6 mice urine vs. female C57BL/6 mice (estrous) urine; (b) Male C57BL/6 mice urine vs. male Balb/c mice urine; (c) Subordinate male C57BL/6 mice urine vs. dominant male C57BL/6 mice urine. The investigation time of the fourth presentation was lower compared with the first presentation (* *P* < 0.05, ** *P* < 0.01, independent-samples *t*-test or Mann-Whitney *U* test). The investigation time of the fifth presentation was higher compared with the fourth presentation of the habituated sample (# *P* < 0.05, independent-samples *t*-test or Mann-Whitney *U* test).

**Fig 2:**
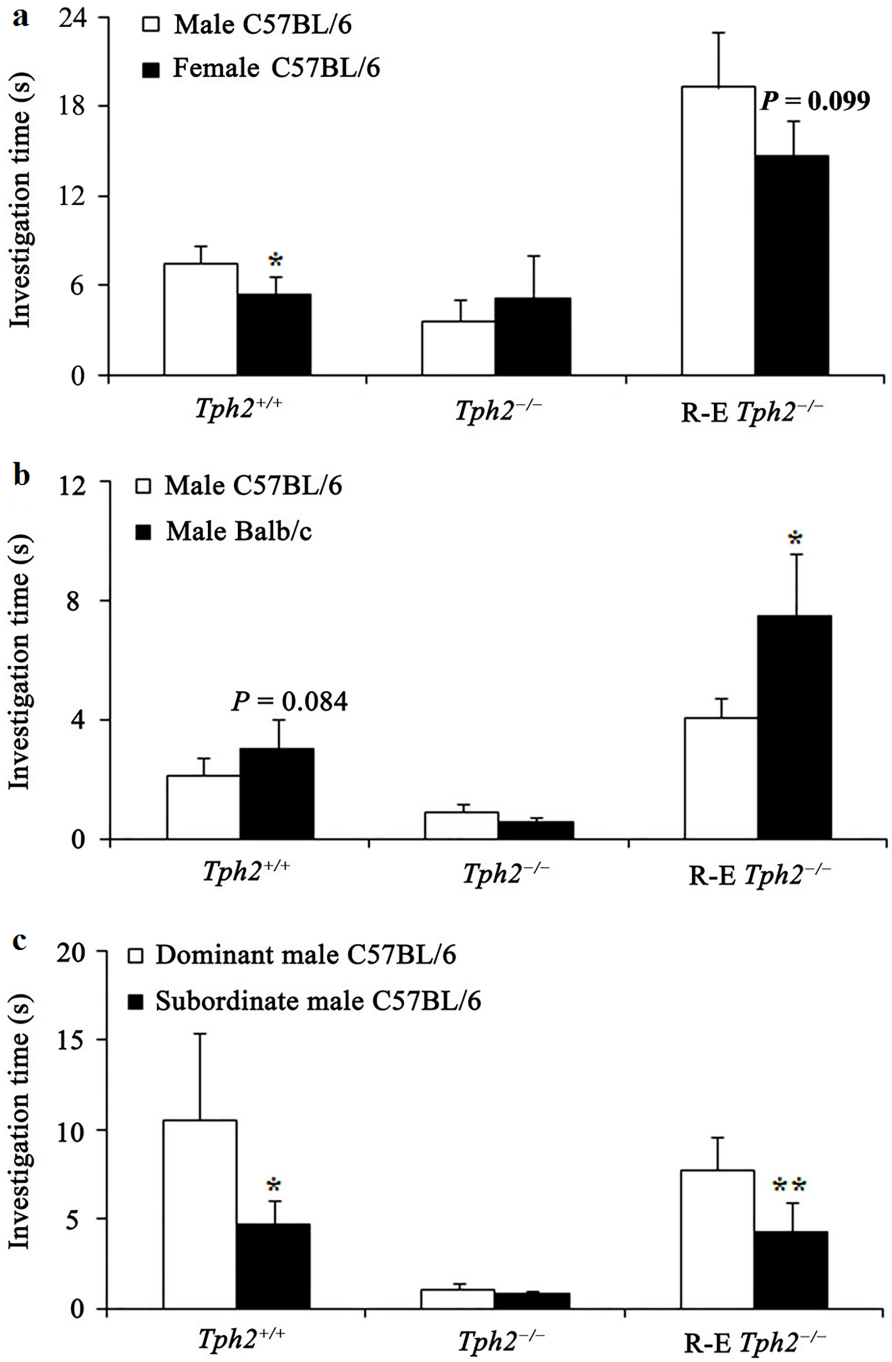
Duration of the investigation (mean ± SE) of 12 sexually naive *Tph2*^*−/−*^ female mice, 12 reproduction-experienced (R-E) *Tph2*^*−/−*^ female mice, and 12 sexually naive *Tph2*^*+/+*^ female mice to urine odor related to sex, genetic relatedness, and social hierarchy during a 3-min choice test. (a) Male C57BL/6 mice urine vs. female C57BL/6 mice (estrous) urine; (b) Male C57BL/6 mice urine vs. male Balb/c mice urine; (c) Dominant male C57BL/6 mice urine vs. subordinate male C57BL/6 mice urine. * *P* < 0.05, ** *P* < 0.01, paired *t*-test or Wilcoxon matched-pairs signed-rank test.

**Fig 3:**
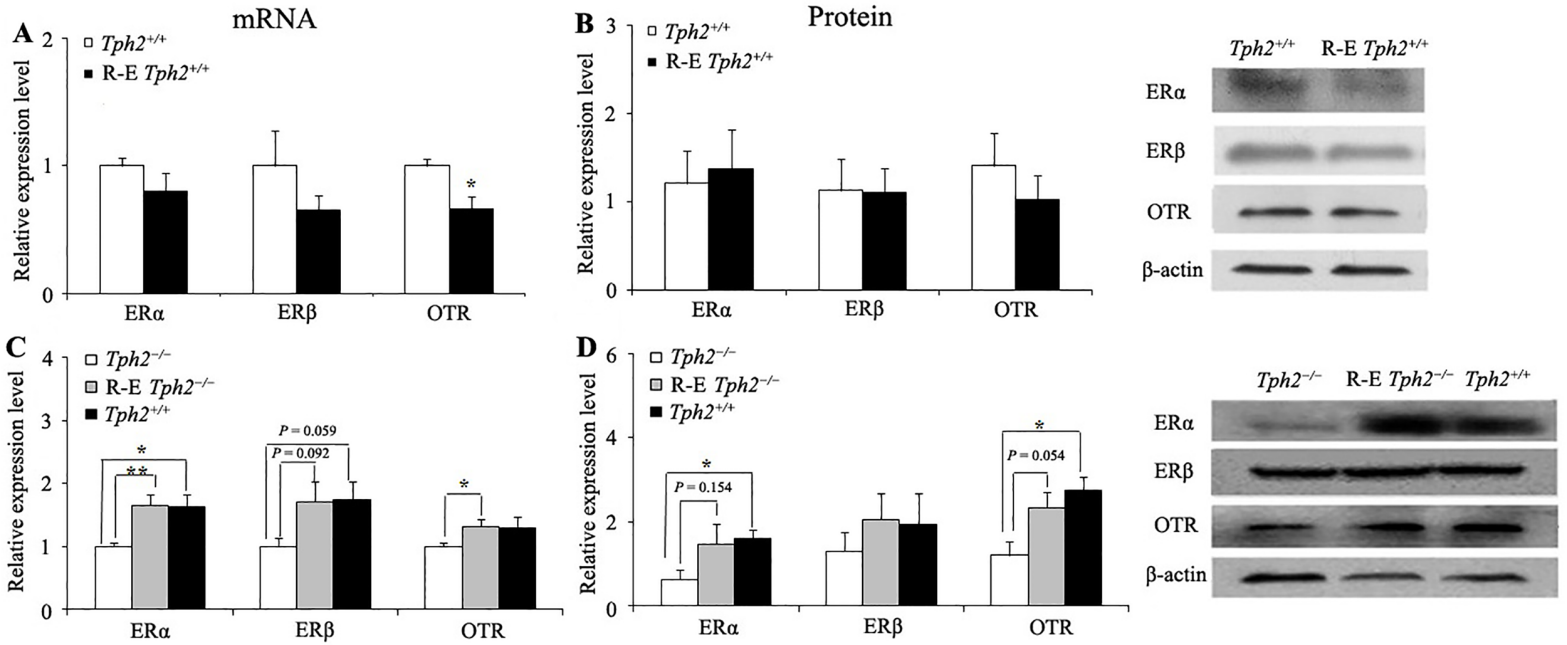
Comparison of the relative expression (mean ± SE) of ERα, ERβ, and OTR in the amygdala at the mRNA and protein level. (a) Comparison of the relative expression of ERα, ERβ, and OTR in the amygdala (unilateral) between sexually naive *Tph2*^*+/+*^ female mice (n = 7) and R-E *Tph2*^*+/+*^ female mice (n = 8) at the mRNA level; (b) Comparison of the relative expression of ERα, ERβ, and OTR in the amygdala (unilateral) between sexually naive *Tph2*^*+/+*^ female mice (n = 7) and R-E *Tph2*^*+/+*^ female mice (n = 7) at the protein level; (c) Comparison of the relative expression of ERα, ERβ, and OTR in the amygdala (bilateral) among sexually naive *Tph2*^*−/−*^ female mice (n = 6), R-E *Tph2*^*−/−*^ female mice (n = 6), and sexually naive *Tph2*^*+/+*^ female mice (n = 6) at the mRNA level; (d) Comparison of the relative expression of ERα, ERβ, and OTR in the amygdala (bilateral) among sexually naive *Tph2*^*−/−*^ female mice (n = 5), R-E *Tph2*^*−/−*^ female mice (n = 5), and sexually naive *Tph2*^*+/+*^ female mice (n = 5) at the protein level. * *P* < 0.05; ** *P* < 0.01, independent-samples *t*-test or Mann-Whitney *U* test.

**Fig 4:**
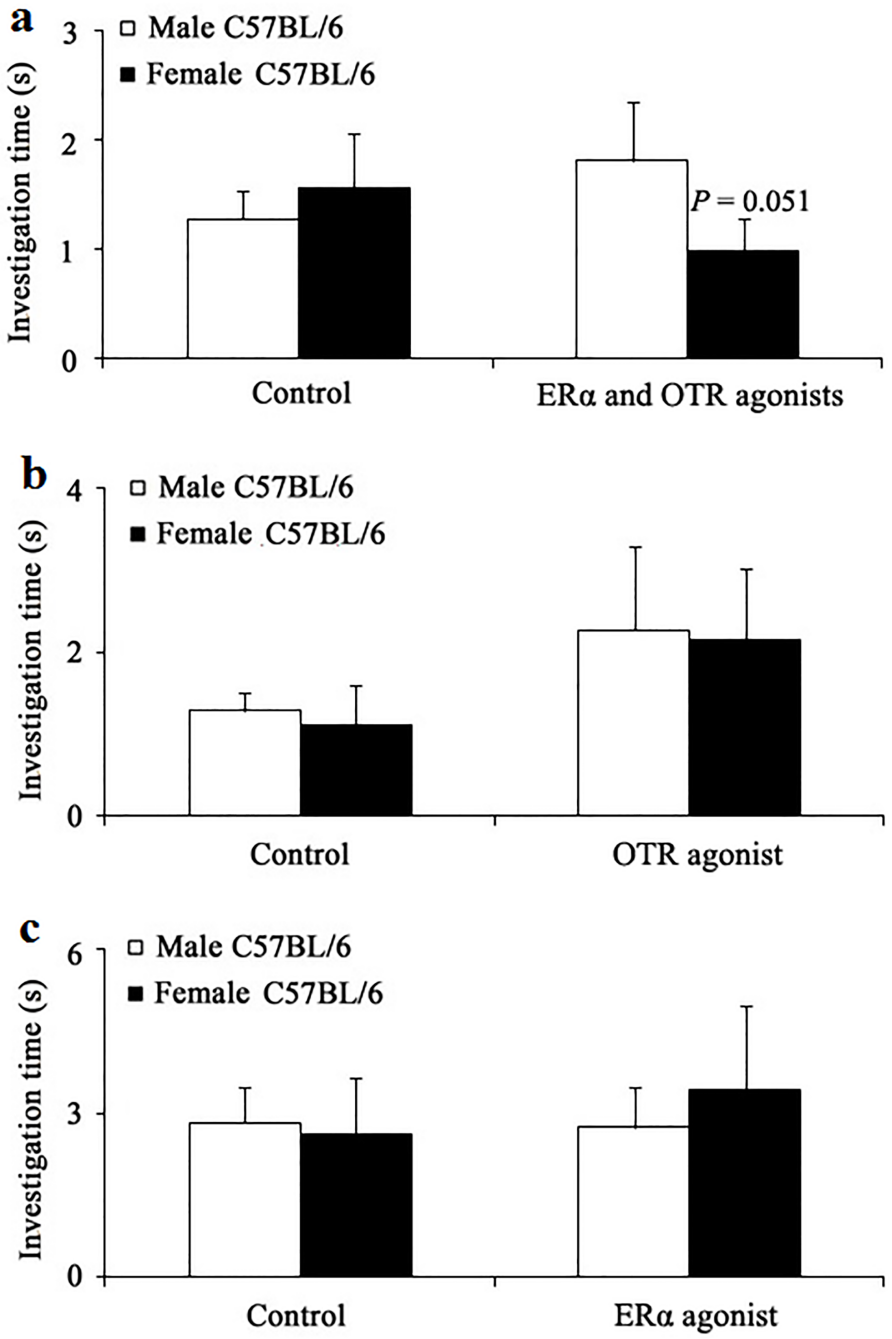
Duration of the investigation (mean ± SE) of sexually naive *Tph2*^*−/−*^ female mice (n _control_ = 7, n _drug_ = 7) to the urine odor of male C57BL/6 mice vs. female C57BL/6 mice (estrous) 30 min after 2 μL drug infusion. Duration of the investigation of sexually naive *Tph2*^*−/−*^ female mice to the urine odor of male C57BL/6 mice vs. female C57BL/6 mice (estrous) after ICV infusion of (a) ERα agonist and OTR agonist in combination for the drug group and 38% DMSO/ACSF for the control group; (b) OTR agonist singly for the drug group and 35% DMSO/ACSF for the control group; (c) ERα agonist singly for the drug group and 38% DMSO/ACSF for the control group. Paired *t*-test or Wilcoxon matched-pairs signed-rank test.

As it was difficult to maintain the *Tph2* line, female mice used in these binary tests were taken from different generations. In Figs 1, 2 and 3, the batch of sexually naive *Tph2*^*−/−*^ female mice and sexually naive *Tph2*^*+/+*^ female mice was Generation 1 (at the age of 12–14 weeks). The batch of reproduction-experienced *Tph2*^*−/−*^ female mice was Generation 2 (at the age of 20–21 weeks). The order of these binary tests was as follows: 1) male C57BL/6 mice urine vs. female C57BL/6 mice (estrous) urine; 2) dominant male C57BL/6 mice urine vs. subordinate male C57BL/6 mice urine; 3) male C57BL/6 mice urine vs. male Balb/c mice urine. Each test recipient female mouse was given 1 d of rest between each test for repeated use. In Fig 4, the batch of 15 sexually naive *Tph2*^*−/−*^ female mice used for intracerebroventricular injection was Generation 3 (at the age of 24–36 weeks). Each of the mice was given approximately 1 week of rest before the next drug administration and the following binary test for repeated use.

### Habituation–dishabituation test

Habituation–dishabituation tests were conducted via one capillary using sexually naive *Tph2*^*−/−*^ female mice (Generation 1, after the binary tests above, at the age of 14–15 weeks) in order to investigate olfactory discrimination ability [3, 29]. In order to induce habituation, we provided each female mouse with one urine odor on a series of four trials and then introduced the other urine odor on the fifth trial. Each trial performed as described previously lasted for 3 min with a 2-min interval between consecutive trials. The order of these tests was as follows: 1) male C57BL/6 mice urine vs. male Balb/c mice urine; 2) male C57BL/6 mice urine vs. female C57BL/6 mice (estrous) urine; 3) subordinate male C57BL/6 mice urine vs. dominant male C57BL/6 mice urine. All tests were conducted in the home cages of the mice during the dark phase, and 1 d of rest was given between each test for repeated use.

### Tissue sampling

After all of the behavioral tests described above, the amygdala (approximately from −0.22 mm to −2.54 mm relative to the bregma) of all the female mice were dissected in a mouse brain matrix on ice [6]. The tissues were immediately frozen in liquid nitrogen and stored at −80 °C until use.

### Real-time polymerase chain reactions

Quantitative real-time PCR was performed, as previously described [6, 30]. Briefly, 2 μg ribonucleic acid (RNA) extracted from the amygdala was reverse-transcribed using a PrimeScript^®^ RT reagent Kit with gDNA Eraser (Perfect Real Time) (Takara), following the manufacturer’s instructions. PCR reactions were performed using RealMasterMix (SYBR Green) (Tiangen, China) using the a Mx3005P quantitative PCR system (Stratagene, La Jolla, CA, USA). Thermal cycling conditions were: 95 °C for 2 min followed by 40 cycles of 95 °C for 20 s, 60 °C for 20 s, and 68 °C for 40 s. To exclude the interference of unspecific products, a melting curve analysis was conducted using high-resolution data collection during an incremental temperature change from 60 to 95 °C with a ramp rate of 0.2 °C /s. *β-actin* was chosen as a reference. Primer sequences were: *β-actin*, forward-*TCCATCATGAAGTGTGACGT*, reverse-*GAGCAATGATCTTGATCTTCAT*; *ERα*, forward-*GCTGGCCTGACTCTGCA*, reverse-*TCTGGCTGGGCTCCTCT*; *ERβ*, forward-*TTGCTCCAGACCTCGTT*, reverse-*CATCTGTCACTGCGTTCA*; *OTR*, forward-*CGTCAATGCGCCCAAAG*, reverse-*CGAGCAGAGCAGCAGAGGAA*. Data were calculated using the 2^-ΔΔ*C*^_*T*_ formula.

### Western blot

Western blot analysis was carried out as previously described [31, 32]. The amygdala was homogenized in lysis buffer (40 μL for unilateral amygdala and 50 μL for bilateral amygdala) comprised of 50 mM tris(hydroxymethyl)aminomethane hydrochloride (Tris-HCl) at pH 8.0, 0.15 M NaCl, 1% Triton X-100, 0.25% sodium deoxycholate, and 4% protease inhibitor cocktail (Roche). Protein concentration was tested using the Bradford protein assay method. Proteins were separated using sodium dodecyl sulfate polyacrylamide gel electrophoresis (SDS-PAGE) and then transferred onto apolyvinylidene difluoride (PVDF) membrane (Beijing Dingguo Changsheng Biotechnology Co., Ltd, China). The membrane was blocked with 5% milk for 1.5 h at room temperature after being washed three times in tris-buffered saline with Tween 20 (TBST; 8% NaCl, 0.2 M Tris-HCl, pH7.5, 1% Tween20) for 10 min each. Incubation with the primary antibody of ERα (1:2000, ab2746, Abcam), ERβ (1:1000, ab3577, Abcam), OTR (1:250, AP17658a, ABGENT), and β-actin (1:5000, cw0263, Beijing CoWin Biotech, China) diluted in blocking buffer was carried out overnight at 4°C. Then, the membrane was washed three times in TBST, for 10 min each, and incubated for 1 h at room temperature with the corresponding horseradish peroxidase (HRP) conjugated secondary antibody (cw0102 or cw0103, Beijing CoWin Biotech, China) diluted 1:5000 in blocking buffer. After being washed three times in TBST for 10 min each time, chemiluminescence with substrate (Millipore, USA) was performed using chemiluminescent-sensitive film (Kodak). Relative amounts were analyzed using Quantity One 4.6.2 software, while β-actin was chosen as the reference.

### Intracerebroventricular (ICV) injection

Dimethyl sulfoxide (DMSO; Beijing Chemical Works, China) and artificial cerebrospinal fluid (ACSF) were selected as the solvent. The vehicle and the final concentration of each drug were as given below: ERα agonist-PPT (Tocris, Cat. No. 1426), 38% DMSO/ACSF, 2.5 μg/μL; OTR agonist-WAY 267464 dihydrochloride (Tocris, Cat. No. 3933), 35% DMSO/ACSF, 1.25 μg/μL.

As described previously [33], another 15 sexually naive *Tph2*^*−/−*^ female mice were anesthetized using sodium pentobarbital (80 mg/kg) by intraperitoneal injection and then placed in a stereotaxic frame (68001, RWD Life Science Co., Ltd, China) with blunt ear bars. One stainless-steel guide cannula (diameter, 0.48 mm, RWD Life Science Co., Ltd, China) was implanted into the brain, targeting the lateral ventricle at the following coordinates (mm) anteroposterior (AP)/mediolateral (ML)/dorsoventral (DV): −0.58/−1.0/−2.0, respectively, relative to Bregma [34]. We have checked that such intracerebroventricular administration would make the given solution diffuse to the whole ventricle. Then, the corresponding stylet (diameter, 0.3 mm; RWD Life Science Co., Ltd, China) was inserted into the cannula and removed only for the drug infusion. Mice were given at least 1 week to recover from surgery before testing. Then, each mouse received an injection of 2 μL test compounds at a rate of 300–400 nL/min. Binary choice tests were performed 30 min later. Each drug administration was repeated once to the same mice after approximately 1 week of rest, and the sum of the two investigation times was calculated and presented in the figure. All test recipient female mice were given approximately 1 week of rest between each new drug infusion for repeated use. Drug administration to sexually naive *Tph2*^*−/−*^ female mice was in the following order (the control group received an injection of the same amount of corresponding solvent): 1) ERα agonist plus OTR agonist in combination; 2) OTR agonist; 3) ERα agonist. During the course of this procedure, one sexually naive and two reproduction-experienced *Tph2*^*−/−*^ female mice belonging to the drug group died.

### Statistical evaluation

The Kolmogorov–Smirnov test was used to determine parametric or nonparametric tests. For binary choice tests, normally distributed data were tested using a paired-samples *t*-test, while non-normally distributed data were examined using a Wilcoxon matched-pairs signed-rank test. For others analyses, independent-samples *t*-test was used for normally distributed data and a Mann-Whitney *U* test was used for the nonparametric test. All statistical analyses were conducted using SPSS 16.0 software (SPSS Inc., Chicago, IL) with the critical value of a = 0.05.

## Results

### Sexually naive *Tph2*^*−/−*^ female mice showed normal olfactory discrimination

In habituation–dishabituation tests, sexually naive *Tph2*^*−/−*^ female mice were habituated to repeated exposure to the urine odor of male C57BL/6 mice (Mann-Whitney *U* test, *Z* = 2.889, *N* = 24, *P* = 0.004, Fig 1a), while they increased their investigation of the urine odor of C57BL/6 females (Mann-Whitney *U* test, *Z* = 2.310, *N* = 24, *P* = 0.021, Fig 1a).

Likewise, after sexually naive *Tph2*^*−/−*^ females habituated themselves to the urine odor of C57BL/6 males (Mann-Whitney *U* test, *Z* = 2.824, *N* = 24, *P* = 0.005, Fig 1b), they could be dishabituated by the urine odor of Balb/c males (Mann-Whitney *U* test, *Z* = 2.510, *N* = 24, *P* = 0.012, Fig 1b). This was also found for sexually naive *Tph2*^*−/−*^ female mice between repeated exposures to subordinate male urine (independent-samples *t*-test, *t* = 2.355, df = 22, *P* = 0.028, Fig 1c) and dominant male urine (independent-samples *t*-test, *t* = –2.465, df = 22, *P* = 0.022, Fig 1c).

### Sexually naive *Tph2*^*−/−*^ females exhibited abnormal sociosexual olfactory preferences, but *Tph2*^*+/+*^ females and reproduction-experienced *Tph2*^*−/−*^ females did not

In the binary choice tests, sexually naive *Tph2*^*−/−*^ female mice showed no olfactory preferences between the paired urine odor (Fig 2a–2c).

Nevertheless, sexually naive *Tph2*^*+/+*^ female mice spent more time sniffing urine from male C57BL/6 mice than from female C57BL/6 mice (paired *t* = 2.707, df = 11, *P* = 0.020, Fig 2a). They also exhibited a tendency to prefer the urine of genetically dissimilar Balb/c males to that from genetically similar C57BL/6 males (Wilcoxon rank sum test, *Z* = 1.726, *N* = 12, *P* = 0.084, marginal significance, Fig 2b). Similarly, sexually naive *Tph2*^*+/+*^ female mice showed an olfactory preference for dominant C57BL/6 males vs. subordinate C57BL/6 males (Wilcoxon rank sum test, *Z* = 2.187, *N* = 12, *P* = 0.028, Fig 2c). In addition, reproduction-experienced *Tph2*^*+/+*^ females also exhibited an olfactory preference for male C57BL/6 mice to female C57BL/6 mice (Wilcoxon rank sum test, *Z* = 2.100, *N* = 8, *P* = 0.036, not shown in figure).

Meanwhile, similar to the sexually naive *Tph2*^*+/+*^ female mice, the *Tph2*^*−/−*^ female mice with reproductive experiences preferred urine odor from male C57BL/6 mice to female C57BL/6 mice (Wilcoxon rank sum test, *Z* = 1.647, *N* = 12, *P* = 0.099, marginal significance, Fig 2a), male Balb/c mice to male C57BL/6 mice (Wilcoxon rank sum test, *Z* = 1.961, *N* = 12, *P* = 0.050, Fig 2b) and dominant males to subordinate males (Wilcoxon rank sum test, *Z* = 2.589, *N* = 12, *P* = 0.010, Fig 2c).

### Both ERα and OTR in the amygdala of reproduction-experienced *Tph2*^*−/−*^ females presented higher expression at the mRNA level and an upward tendency at the protein level

Messenger RNA (mRNA) expression (independent-samples *t*-test, *t* = 2.812, df = 12, *P* = 0.016, Fig 3a), but not protein expression (Fig 3b), of OTR in the amygdala was lower in *Tph2*^*+/+*^ females with reproductive experiences as compared with sexually naive *Tph2*^*+/+*^ females. The expression of ERα and ERβ genes did not differ at either the mRNA or protein level between the sexually naive and reproduction-experienced *Tph2*^*+/+*^ females (Fig 3a and 3b).

However, relative to sexually naive *Tph2*^*−/−*^ females, the mRNA expression of ERα (independent-samples *t*-test, *t* = 3.348, df = 10, *P* = 0.007, Fig 3c) and OTR (independent-samples *t*-test, *t* = 2.486, df = 10, *P* = 0.032, Fig 3c) in the amygdala became significantly higher in reproduction-experienced *Tph2*^*−/−*^ females. Similarly, compared with sexually naive *Tph2*^*−/−*^ females, the protein expression of OTR (independent-samples *t*-test, *t* = 2.252, df = 8, *P* = 0.054, marginal significance, Fig 3d) also exhibited an upward tendency in the amygdala of reproduction-experienced *Tph2*^*−/−*^ females. Moreover, there was a clear trend that the protein expression of ERα in the amygdala of reproduction-experienced *Tph2*^*−/−*^ females was higher than in sexually naive *Tph2*^*−/−*^ females (0.6374 ± 0.2385 vs. 1.4734 ± 0.4743, independent-samples *t*-test, *t* = 1.575, df = 8, *P* = 0.154, Fig 3d), although the difference did not reach statistical difference. In addition, as compared with sexually naive *Tph2*^*−/−*^ females, ERβ gene expression in the amygdala of reproduction-experienced *Tph2*^*−/−*^ females presented an upward tendency at the mRNA level (independent-samples *t*-test, *t* = 1.886, df = 9, *P* = 0.092, marginal significance, Fig 3c), but was unaltered at the protein level (Fig 3d).

Furthermore, as compared with sexually naive *Tph2*^*+/+*^ female mice, the mRNA expression of ERα (independent-samples *t*-test, *t* = 2.878, df = 10, *P* = 0.016, Fig 3c) in the amygdala of sexually naive *Tph2*^*−/−*^ females was lower, while the mRNA expression of ERβ also had a downward tendency (independent-samples *t*-test, *t* = 2.159, df = 9, *P* = 0.059, marginal significance, Fig 3c). Likewise, at the protein level, compared with sexually naive *Tph2*^*+/+*^ female mice, the expression of ERα (Mann-Whitney *U* test, *Z* = 2.402, *N* = 10, *P* = 0.016, Fig 3d) and OTR (Mann-Whitney *U* test, *Z* = 2.193, *N* = 10, *P* = 0.028, Fig 3d) rather than ERβ were also lower in the amygdala of sexually naive *Tph2*^*−/−*^ females.

### OTR and ERα agonists combination, but not alone, rescued the deficit in the sexual olfactory preference of sexually naive *Tph2*^*−/−*^ female mice to a degree

Binary choice tests revealed that infusion of OTR and ERα agonists in combination could elicit sexually naive *Tph2*^*−/−*^ females to choose the urine odor of C57BL/6 males over that of C57BL/6 females to some extent (Wilcoxon rank sum test, *Z* = 1.947, *N* = 7, *P* = 0.051, marginal significance, Fig 4a), while the control group showed no sexual preferences (paired *t* = 0.778, df = 6, *P* = 0.466, Fig 4a). However, a single infusion of either OTR agonist or ERα agonist failed to elicit such sexual preferences (Fig 4b and 4c).

## Discussion

It is well known that 5-HT is closely related to central information processing, stress adaption, anxiety, depression, aggression, sexual preference, and maternal care in rodents [14, 15, 18, 19, 35, 36]. The current study, in which sexually naive *Tph2*^*−/−*^ female mice could not recognize urine odor related to sex, corroborated previous studies that deficiency in central 5-HT could bring the loss of sexual preference [14, 15]. Additionally, the findings that sexually naive *Tph2*^*−/−*^ female mice could not recognize urine odor not only related to sex but also related to genetic relatedness and social hierarchy despite possessing normal olfactory discrimination extended previous observations [14, 15, 37].

As evident from extensive literature in this area, sexual preference can be affected by previous sexual experiences in both early life and adulthood in many species of rodents, such as hamsters, lemmings, prairie voles, and mice [8, 9, 38, 39, 40, 41, 42]. Usually, previous sex-related experiences would lower the threshold for stimuli that is capable of eliciting copulatory behaviors, increase sexual receptivity, confer a reproductive advantage, and increase the probability of coming into contact with better quality mates [8, 43, 44, 45, 46]. Furthermore, reproductive experiences have been suggested as playing a role in altering the endocrine status and conferring beneficial changes to females in terms of improving cognition and enhancing some aspects of memory in sexual behavior [10, 11]. For example, lesioning the vomeronasal organ (VNO), which is mainly responsible for the perception of sex pheromones, induced a loss of sexual responses in sexually naive males; however, sexual experiences restored such impairment by activating the main olfactory system [13, 47, 48, 49]. In the current study, reproductive experiences mitigated the disruptive effect of *Tph2* knockout and may, thereby, have guided *Tph2*^*−/−*^ female mice toward male urine odor.

Reproduction-experienced *Tph2*^*−/−*^ female mice gained sexual olfactory preference, implying that some facilitative mechanisms may exist. The pathways through which estrogens and serotonin exert their effects appear to be linked, although each has an independent effect [22, 23]. A four-gene micronet involving ERα, ERβ, OT, and OTR has been proposed as the underlying regulatory basis [24, 50]. In the central processing of odorant signals, the amygdala is one of the most important brain regions, where olfactory signals from both the main and accessory olfactory system finally converge to enable socio-sexual recognition [2, 50, 51, 52]. Here, we found that the expressions of ERα and OTR in the amygdala of reproduction-experienced *Tph2*^*−/−*^ female mice were higher than in sexually naive *Tph2*^*−/−*^ females at the mRNA level. Meanwhile, at the protein level, relative to the sexually naive *Tph2*^*−/−*^ females, the expressions of ERα and OTR in the amygdala of reproduction-experienced *Tph2*^*−/−*^ female mice also exhibited an upward tendency. Moreover, OTR and ERα agonists in combination, but not singly, had a tendency to rescue the sexual olfactory preference of sexually naive *Tph2*^*−/−*^ female mice to some degree.

Consequently, from the upregulated expression of ERα and OTR in the amygdala of reproduction-experienced *Tph2*^*−/−*^ female mice together with the pharmacological effect of ERα and/or OTR agonist on sexual olfactory preference of *Tph2*^*−/−*^ female mice, we speculate that ERα and OTR might act in a combined way to exert their effects. Such findings confirm and further expand on the view of Young that ERα and OTR in the amygdala are necessary for the regulation of social identification and recognition [27]. Furthermore, these findings suggest that ERα and OTR might constitute the neurobiological basis through which estrogens and the OT system affect the central 5-HT system in its mediation of olfactory preference. Additionally, ERα and ERβ may differ in their importance for neuroprotection in that ERα, rather than ERβ, might be the required receptor for the neuroprotective effect following brain injury [53]. The present results also indicate a differential role of ERα and ERβ in regulating sexual olfactory preference in the context of a defective 5-HT signaling system in the brain.

However, as stated above, it should be pointed out that there were some weaknesses in the current study. As it is difficult to maintain the *Tph2* line, we only have a limited sample size for each batch and each test. This small sample size may have caused some results to present marginal significance or no statistical difference, even though the tendency was suggestive. Furthermore, the fact that the estrous cycles were not determined in any test for recipient female mice was really a limitation and might account for the high variability and lack of statistical significance for some outcomes. Also, different batches used in binary choice tests was another limitation and might be the most important reasons that the investigation time seem to vary considerably between reproduction-experienced *Tph2*^*−/−*^ female mice in Figs 2 and 4. Moreover, restricted by technical limitations, for gene expression analysis, we just investigated the whole amygdala and could not identify a precise location of the specific subnuclei. Likewise, the ICV infusion just referred to the lateral ventricle rather than any specific brain area. Thus, this research remained at the level of rough correlation analysis and should be regarded with caution.

## Conclusions

In conclusion, our results indicate that *Tph2* knockout affected the ability of olfactory recognition rather than olfactory discrimination, which confirms and expand on previous findings. For the first time, reproductive experiences have been shown to mitigate the disruptive effect of *Tph2* knockout on the sexual olfactory preference of female mice. Furthermore, ERα and OTR activation might constitute the neural substrates that are involved in the facilitative mechanisms.
