# Correction: *Tph2^−/−^* female mice restore socio-sexual recognition through upregulating ERα and OTR genes in the amygdala

**DOI:** 10.1371/journal.pone.0195245

**Published:** 2018-03-27

**Authors:** Ying Huo, Yaohua Zhang, Huifen Guo, Yingjuan Liu, Qi Fang, Jianxu Zhang

There is an error in the first sentence of the Abstract. The correct sentence is as follows: The central 5-hydroxytryptamine deficiency impairs sociosexual behaviors and olfaction preferences in sexually naive mice.
